# Global observational coverage of onshore oil and gas methane sources with TROPOMI

**DOI:** 10.1038/s41598-023-41914-8

**Published:** 2023-10-05

**Authors:** Mozhou Gao, Zhenyu Xing, Coleman Vollrath, Chris H. Hugenholtz, Thomas E. Barchyn

**Affiliations:** Centre for Smart Emissions Sensing Technologies, Department of Geography, University of Calgary, 2500 University Drive NW, Calgary, AB T2N 1N4 Canada

**Keywords:** Environmental sciences, Environmental social sciences

## Abstract

Satellite observations have been used to measure methane (CH_4_) emissions from the oil and gas (O&G) industry, particularly by revealing previously undocumented, very large emission events and basin-level emission estimates. However, most satellite systems use passive remote sensing to retrieve CH_4_ mixing ratios, which is sensitive to sunlight, earth surface properties, and atmospheric conditions. Accordingly, the reliability of satellites for routine CH_4_ emissions monitoring varies across the globe. To better understand the potentials and limitations of routine monitoring of CH_4_ emissions with satellites, we investigated the global observational coverage of the TROPOMI instrument onboard the Sentinel-5P satellite—the only satellite system currently with daily global coverage. A 0.1° × 0.1° gridded global map that indicates the average number of days with valid observations from TROPOMI for 2019–2021 was generated by following the measurement retrieval quality-assurance threshold (≥ 0.5). We found TROPOMI had promising observational coverage over dryland regions (maximum: 58.6%) but limited coverage over tropical regions and high latitudes (minimum: 0%). Cloud cover and solar zenith angle were the primary factors affecting observational coverage at high latitudes, while aerosol optical thickness was the primary factor over dryland regions. To further assess the country-level reliability of satellites for detecting and quantifying CH_4_ emissions from the onshore O&G sector, we extracted the average annual TROPOMI observational coverage (TOC) over onshore O&G infrastructure for 160 countries. Seven of the top-10 O&G-producing countries had an average annual TOC < 10% (< 36 days per year), which indicates the limited ability to routinely identify large emissions events, track their duration, and quantify emissions rates using inverse modelling. We further assessed the potential performance of the latter by combining TOC and the uncertainties from the global O&G inventory. Results indicate that the accuracy of emissions quantifications of onshore O&G sources using TROPOMI data and inverse modeling will be higher in countries located in dryland and mid-latitude regions and lower in tropical and high-latitude regions. Therefore, current passive-sensing satellites have low potential for frequent monitoring of large methane emissions from O&G sectors in countries located in tropical and high latitudes (e.g., Canada, Russia, Brazil, Norway, and Venezuela). Alternative methods should be considered for routine emissions monitoring in these regions.

## Introduction

Methane (CH_4_) is a potent greenhouse gas (GHG) with 84 times the global warming potential of CO_2_ over a 20-year period^1^. Atmospheric CH_4_ concentrations have increased 260% since the pre-industrial era; an increase that is primarily due to human activities^2,3^. Reducing CH_4_ emissions is internationally recognized as the most effective strategy to mitigate near-term global warming^4^. Following the United Nations Framework Convention on Climate Change (UNFCCC) Conference of the Parties 26 (COP26), more than 100 countries committed to the Global CH_4_ Pledge—a voluntary international initiative targeting a 30% reduction in CH_4_ emissions across all economic sectors in participating countries by 2030. Emissions reduction goals are becoming more ambitious and regulations more stringent. As such, it is necessary to evaluate annual progress towards CH_4_ emissions reduction targets and monitoring emission sources at global scales.

Satellites can play a role in detecting large emission events and estimating emissions on global and regional scales^5,6^ Most satellite instruments measure CH_4_ passively in the shortwave infrared (SWIR), relying on the absorption characteristics of CH_4_ at 1650 and 2300 nm^5^. With appropriate satellite retrieval methods, SWIR radiation can be used to infer column-integrated CH_4_ mixing ratios^5,6^. In the past two decades, many studies have estimated CH_4_ emissions rates using various satellite systems, such as Sentinel-2, Sentinel-5P, GOSAT, GHGSat, PRISMA, Gaofen5, Ziyuan1^7–26^. Due to the growing interest in monitoring CH_4_ emissions from space through initiatives like the UN’s Methane Alert and Response System (MARS), many satellites originally designed for different purposes but with effective ranges to detect CH_4_ in the SWIR are now being used for this application. This includes systems such as PRISMA, Landsat-8, Worldview-3, and Sentinel-2^15,17,27^.

The Tropospheric Monitoring Instrument (TROPOMI) onboard Sentinel-5 Precursor currently outperforms other satellite instruments with the shortest revisit time (less than 24 h) and global coverage up to 5.5 × 3.5 km^2^ pixel resolution. Thus, it has become a popular satellite in the past five years to quantify regional/basin-scale emissions, reconcile bottom-up CH_4_ emission inventories, validate annual emission estimates from different sectors, and identify ultra-emitter events^11,14,20,22–24,26,28,29^. However, due to coarse spatial resolution, it often cannot locate the precise source that is emitting. Like other passive satellite instruments, the actual performance and observational coverage of TROPOMI are limited by the instrument parameters, artifacts of the atmospheric components, and land surface characteristics^30,31^.

While many more methane-sensing satellites will be launched into space in the next decade, it remains unknown how they will advance global CH_4_ emissions monitoring. Understanding the global observational coverage of TROPOMI reveals geographical challenges for monitoring CH_4_ emissions and factors that are likely to affect the performance of similar satellites (i.e., polar-orbiting sun-synchronized, low-earth orbiting satellites with passive sensing systems). This global analysis addresses three questions: First, where and when does TROPOMI have the most (and least) valid observations? Second, which measurement-impeding factor is the coverage of TROPOMI most sensitive to? Third, how reliable is TROPOMI for different emissions applications? Answers to these questions will also provide insight into the potential observational performance of planned CH_4_ emissions monitoring satellites. To answer these questions, we investigated the global observational coverage of TROPOMI data products to understand the geographical distribution of valid observations for CH_4_ emission detection and quantification.

## Results

### Global TROPOMI observational coverage (TOC)

**Figure 1:**
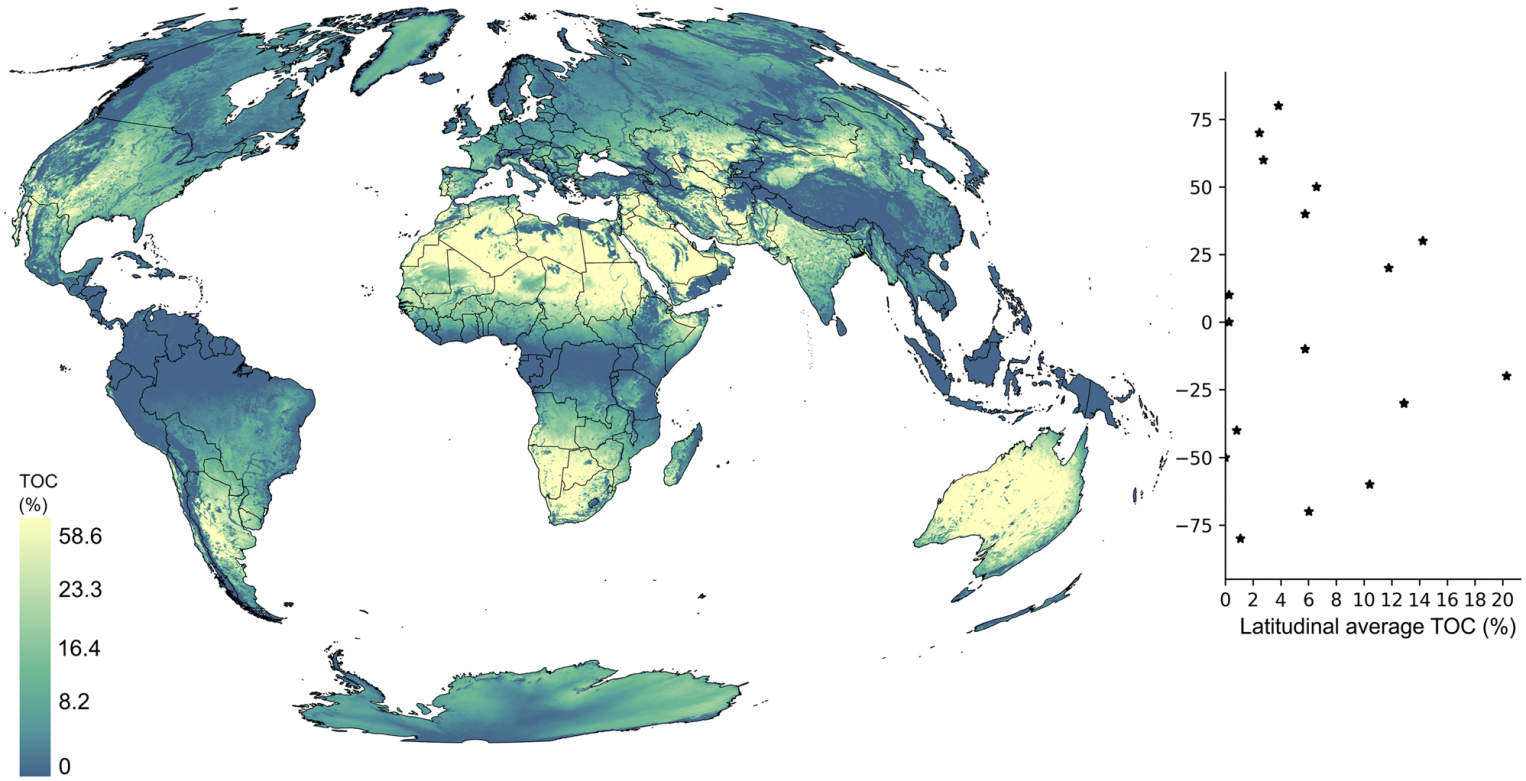
Average annual TOC between 2019 and 2021. The scatter plot on the left shows the average TOC by latitude over land. The map was created using ArcGIS Pro 3.1.2 (https://www.esri.com/en-us/arcgis/products/arcgis-pro/overview).

Figure 1 shows the global distribution of 3-year average global TROPOMI observational coverage (TOC) over land between 2019 and 2021. The global mean and median TOCs are 6.6% and 3.8% over land, respectively. The global pattern indicates the highest values are over the subtropics and mid-latitude dryland (arid to sub-humid) regions and the lowest values over high-latitude regions and near the equator (Fig. 1). These patterns are similar to the number of valid observations found in past studies^6,24^. There are almost no valid observations from TROPOMI over tropical regions along the equator throughout the year. Low TOC is also found over regions with high-elevation terrain and orographic features, such as the Rocky Mountains in North America, the Andes in South America, and the Tibetan Plateau in Asia. Overall, Fig. 1 outlines the climate and topographic conditions that are favorable and less favorable for applying TROPOMI (and other similar satellites) to monitor CH_4_ emissions.

### Seasonal variability of TOC

**Figure 2:**
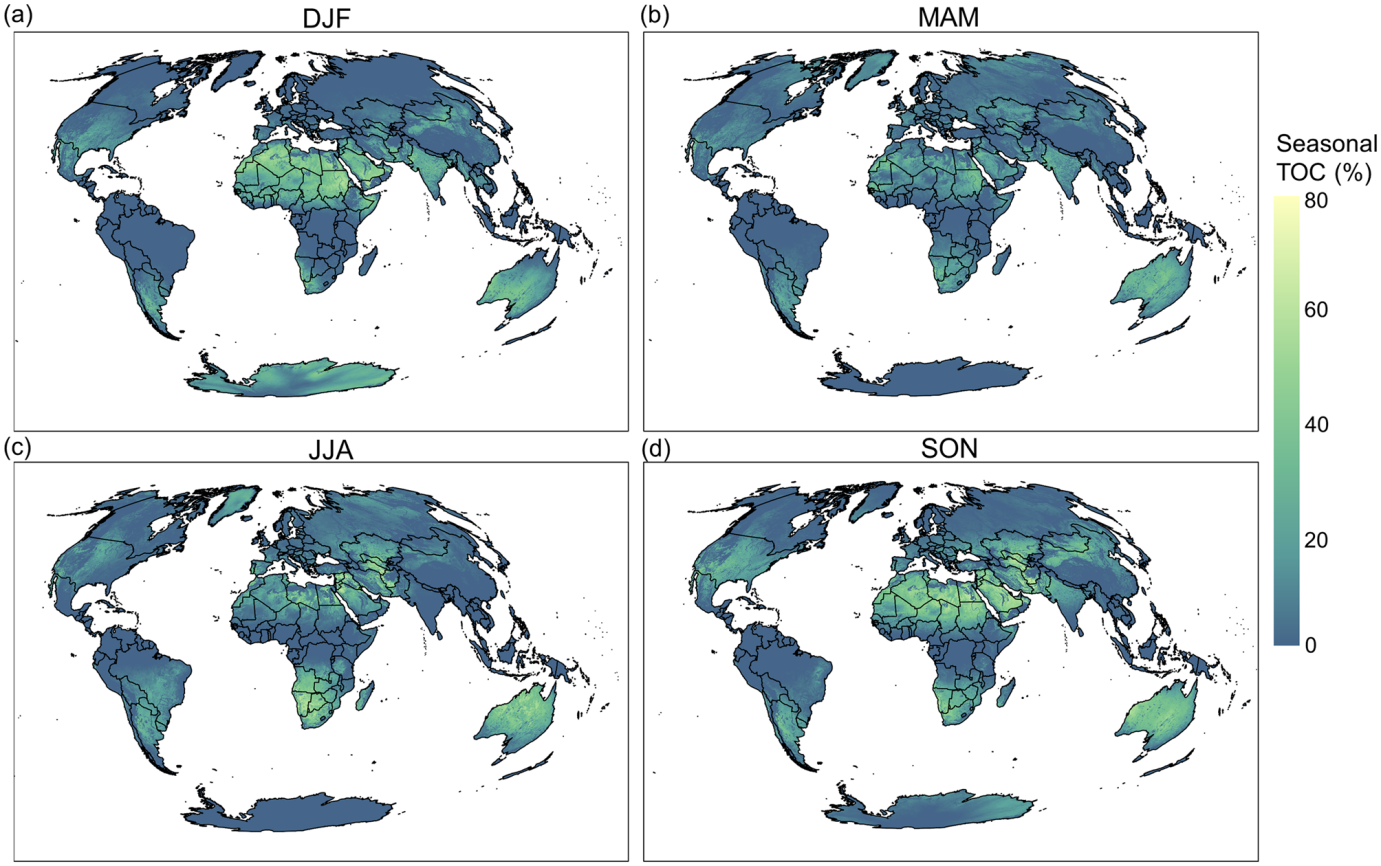
Average TOC by season between 2019–2021: (**a**) DJF December–January–February, (**b**) MAM March–April–May, (**c**) JJA June–July–August, (**d**) SON September–October–November. The map was created using ArcGIS Pro 3.1.2 (https://www.esri.com/en-us/arcgis/products/arcgis-pro/overview).

Seasonal variations of the average TOC over different regions are shown in Fig. 2. The average seasonal TOC is lowest in MAM (4.6%) and highest in SON (7.7%). In all four seasons, TOC is very low in a ~ 20° latitudinal zone centered at the Intertropical Convergence Zone (ITCZ) and at high latitudes except for Greenland in JJA and Antarctica in DJF when anticyclones establish over those regions. TOC is near zero above 50°N in DJF, including most of Canada, Europe, and Russia, and there is a very marginal increase in TOC over these regions in the other seasons. Globally, TOC is highest over Northern Africa (Sahel and Sahara) and the Middle East in SON and DJF but decreases in MAM and remains low over Northern Africa and southern portions of the Middle East into JJA. Seasonal variations of TOC over Northern Africa coincide with shifts in the ITCZ and resulting effects from the African monsoons and increased dust loading in MAM and JJA^32,33^. TOC over Southern Africa is highest in JJA and lowest in DJF, which coincides with changes in the position of the ITCZ and associated monsoons. Over Central Asia and Australia, TOC is highest in SON and lowest in DJF.

### TOC-reducing factors

**Figure 3:**
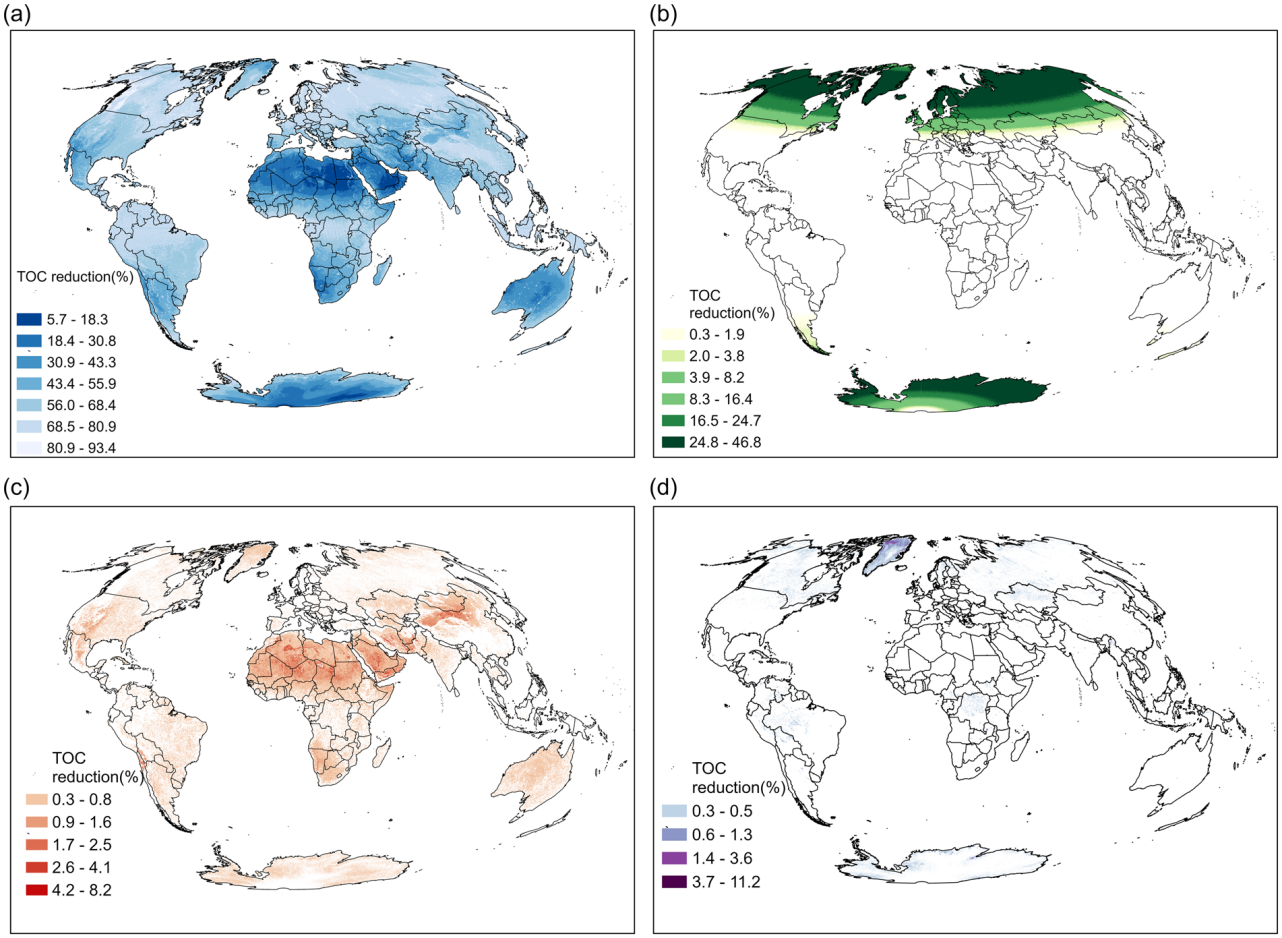
Average annual reduction (%) of TOC by factor for the period 2019–2021: (**a**) cloud cover fraction, (**b**) SZA, (**c**) AOT (NIR), and (**d**) surface albedo (SWIR). The map was created using ArcGIS Pro 3.1.2 (https://www.esri.com/en-us/arcgis/products/arcgis-pro/overview).

Surface characteristics (topography, surface albedo), atmospheric conditions (clouds and aerosols), and solar geometry (solar zenith angle—SZA) are the main factors that influence the availability of observations and the quality of XCH_4_ retrievals. Topography is represented by surface roughness (i.e., standard deviation of altitude based on Global Multi-resolution Terrain Elevation Data (GMTED2010))^34^. Mountainous areas with surface roughness > 100 m are excluded prior to the retrieval of XCH_4_. Results from our analysis of global surface roughness (Figure S1) show that there is an association between mountainous areas and regions with the lowest TOC. Excluding topography, the effects of surface albedo, atmospheric aerosols, and solar geometry on TOC are shown in Fig. 3. Cloud cover has the largest impact on TOC, with a global average annual reduction of 40.0% (146 days), followed by SZA (11.5% or 42 days), aerosol optical thickness (AOT; 0.07% or 0.27 days), and surface albedo (0.04% or 0.13 days). Approximately 79.3% of land-based regions are impacted by persistent cloud cover for at least 90 days (24.7%), with several regions experiencing persistent cloud cover for more than 240 days (66%; e.g., high latitudes, Southeast Asia, Amazon rainforest, and Tibetan Plateau). The effects of both cloud cover and SZA are pronounced over broad swaths of Canada and Russia—two of the largest oil and gas producing countries in the world. In boreal winter, high SZA at high northern latitudes results in no valid observations for up to 3 months. Surface albedo has the smallest impact on TOC globally and is mostly influential only in Greenland. AOT has the most pronounced effects on TOC over major desert regions, such as the Sahara, Arabian, Taklimakan, and Gobi, which is expected as these are major source regions for the global dust cycle^32^.

### TOC for monitoring CH_4_ emissions from the O&G sector

**Figure 4:**
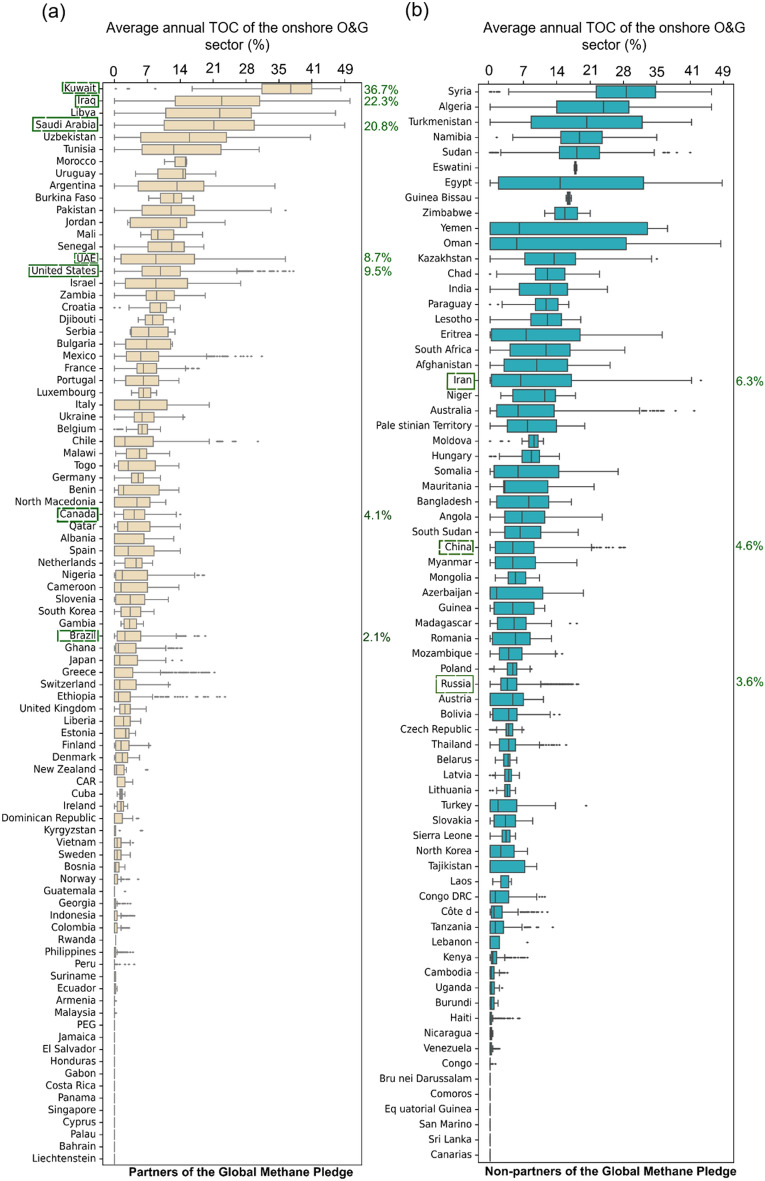
Average annual TOC of onshore O&G infrastructure160 countries ranked by median: (**a**) 89 partners to the Global Methane Pledge, and (**b**) 71 non-partners to the Global Methane Pledge (after COP26). The top-10 producing countries based on total energy production from petroleum and other liquids 2021^35^ are outlined in green. Note that the highest producers may not be the highest emitters. The median average annual TOCs of highlighted countries are shown on the right of the boxplots.

Based on the global variation of TOC in Fig. 1, we investigated the potential of TROPOMI for monitoring CH_4_ emissions from onshore O&G infrastructure in 160 countries across the world. The boxplots in Fig. 4 show distributions of average annual TOC over O&G infrastructure in partner (*n* = 89) and non-partner (*n* = 71) countries of the Global Methane Pledge (after COP26). Some countries have large regional differences in TOC, as expressed by wide interquartile ranges and whiskers (e.g., Oman), while others have very small differences (e.g., Norway). Most of the 160 countries have relatively low median average annual TOC (< 15%) over their O&G infrastructure. In aggregate, non-partner countries have a higher median average annual TOC (4.7%) compared to partner countries (1.9%).

For the onshore O&G infrastructure in each country in Fig. 4, we calculated the median 3-year average number of consecutive days and gap days with valid observations (see Supplementary Table S2). This provides an indication of the ability to monitor large emissions events and determine when they begin and end, which helps investigate the source, determine the total mass or volume of the emission, and potentially determine penalties or fees. As an example, Kuwait's onshore O&G infrastructure has the highest median average TOC (36.7%), highest median consecutive number days with valid observations (3 days), and the fewest median gap days (4 days). Results for the remaining top-10 O&G producing countries^35^ are as follows: USA (9.5%, 2 days, 15 days), Russia (3.6%, 2 days, 37 days), Saudi Arabia (20.8%, 3 days, 8 days), Canada (4%, 2 days, 31 days), Iraq (22.3%, 3 days, 8 days), China (4.6%, 1 day, 42 days), UAE (9%, 2 days, 21 days), Iran (6% 2 days, 35 days), and Brazil (2%, 1 day, 90 days). Some of these countries represent reasonable targets for routine monitoring, particularly those located in dryland regions (Kuwait, Saudi Arabia, Iraq, and UAE), while monitoring capabilities in tropical (Brazil) or high latitude countries are very limited (Russia and Canada). It is worth noting that Iran is in a dryland region but has low TOC because most of its onshore O&G infrastructure is in regions with undulating topography (see Supplementary Fig. S1).

The median of average annual TOCs for the onshore O&G sectors in the USA and China were relatively low because the O&G infrastructure in these countries are spatially distributed across different latitudes and climate zones. Low TOC in Russia and Canada is mainly a result of high cloud cover and high SZA in boreal winter. The differences in TOCs among the top-10 producing countries indicates not all countries receive equal coverage of potential sources, which could create an observational bias where countries with high TOC receive more attention in the scientific literature and associated media. For example, large emissions in Algeria (23.2%, 3 days, 7 days) and Turkmenistan (20%, 3 days, 9 days) have been featured in many peer-reviewed publications since 2017^9,12,13,15,17,25,36^, whereas large emissions from countries like Venezuela (0%, 0 day, 304 day), which holds the largest crude oil reserves in the world, are largely unknown based on satellite data due to persistent cloud cover. The ability to apply passive satellite remote sensing to detect large emissions quickly is therefore unequal among major O&G producing countries.

It should be noted that the emissions source rate and meteorological conditions may also influence CH_4_ emissions detection and quantification with TROPOMI. Large plumes and high XCH_4_ enhancements associated with “ultra-emitters” (> 25 tons/hour) from the onshore O&G sector will have a higher probability of detection compared to smaller emissions given the same TOC conditions, particularly at low turbulence intensities^24^. Thus, for countries and regions with frequent or persistent ultra-emitters, low TOC may still reveal segments or the full extent of large plumes. For instance, while average annual TOCs for Iran and Russia were calculated as 6% and 3.6%, respectively, results from Lauvaux et al.^24^ show the potential of TROPOMI to detect and quantify onshore O&G CH_4_ ultra-emitters over these countries. However, regular and on-demand monitoring of emissions events will be less reliable.

### Potential effect of TOC on regional CH_4_ emission quantification

**Figure 5:**
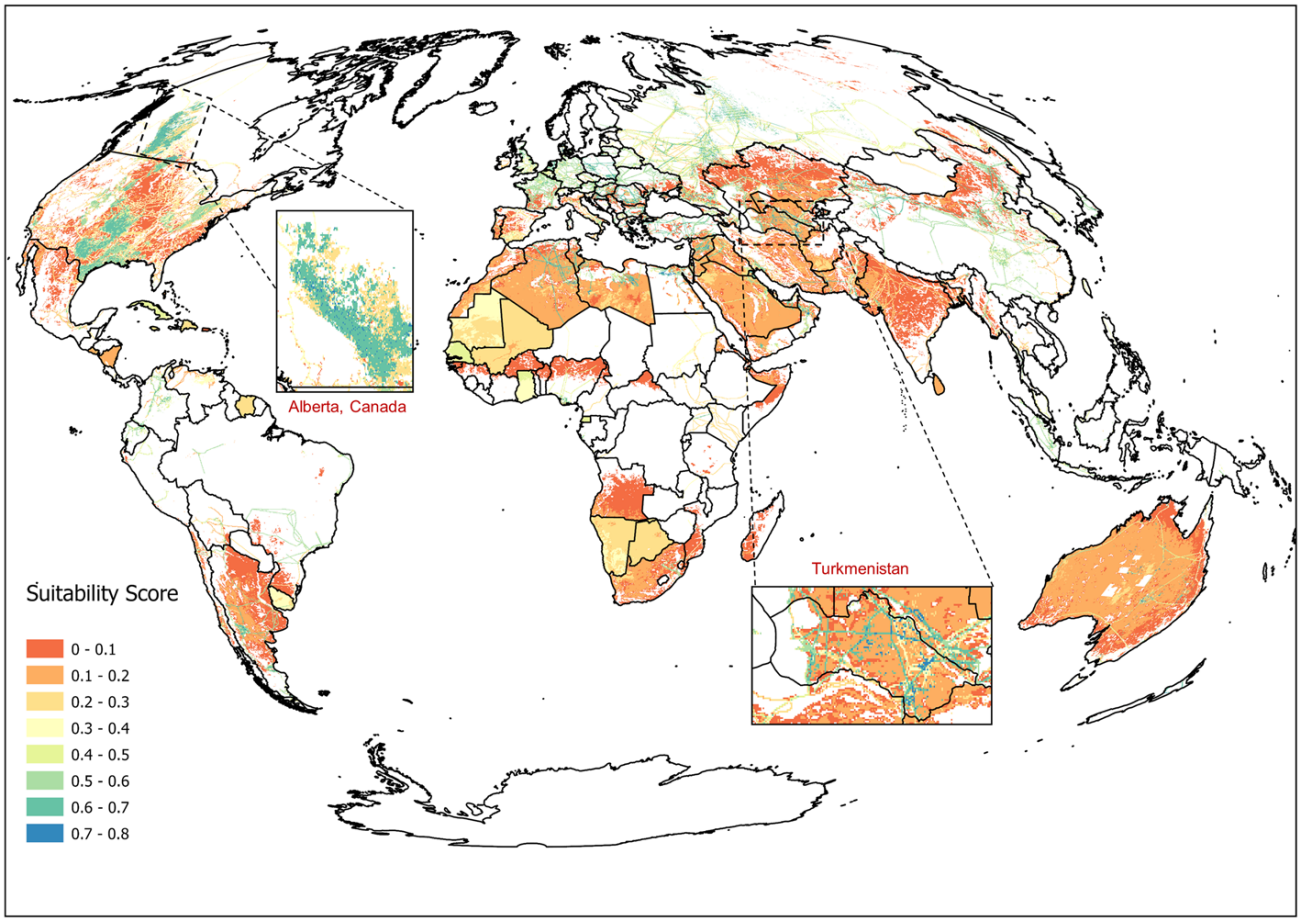
Global suitability scores of using atmospheric inverse modelling to quantify regional CH_4_ emissions from onshore O&G production regions. Turkmenistan is highlighted because multiple O&G producing regions had high suitability scores (> 0.7). Alberta, Canada is highlighted because multiple O&G producing regions had moderate suitability scores (between 0.5 and 0.7) although it had low TOC. Countries with no detailed geographical locations of onshore O&G emission sources in GFEI were represented using sold colors. It is worth nothing that errors in GFEI were carried over to the result of suitability scores. The map was created using ArcGIS Pro 3.1.2 (https://www.esri.com/en-us/arcgis/products/arcgis-pro/overview).

Figure 5 shows the suitability scores of atmospheric inverse modeling for global onshore O&G infrastructure. Most onshore O&G producing regions have moderate suitability scores (> 0.5), such as the Permian Basin in the U.S., the Tarim Basin, China, and Hassi R’Mel, Algeria. Therefore, emissions estimates from atmospheric inverse modelling over these regions can be used to infer actual emission rates, although uncertainties may exist. Grid cells in O&G producing regions at high latitudes, such as the Western Canadian Sedimentary Basin (Canada) and the West Siberian Petroleum Basin (Russia), also have moderate suitability scores, which is primarily because of low relative uncertainties (see Supplementary Fig. S2) in the Global Fuel Exploitation Inventory (GFEI). Nonetheless, there are many grid cells in these two regions with low suitability scores (< 0.3) due to the low TOC. Following the global TOC distribution, consistently low suitability scores (< 0.3) were found for several O&G producing countries near the equator such as Venezuela, Kenya, and Nigeria. This demonstrates the limits of TROPOMI in quantifying emissions with inverse methods for some large O&G basins. High suitability scores (> 0.7) were found along pipelines and over multiple O&G producing regions in Turkmenistan.

This is due to the combined effect of low uncertainties in the bottom-up (BU) inventory and high TOCs. Overall, our analysis indicates methods like the Integrated Methane Inversion 1.0^29^ is not a universal solution to estimating regional emissions. As such, reconciling emissions estimates to BU inventories should not rely exclusively on TROPOMI in O&G sectors with low suitability such as Canada, Russia, and some countries in South America, Africa, and Europe.

## Discussion

Satellite systems have enhanced the ability to detect and quantify high magnitude (“super” or “ultra”) CH_4_ emissions events globally from the O&G sector, as well as other large anthropogenic sources like landfills^37^ and are helping shape strategies and initiatives to respond and ultimately reduce those emissions^38^. Until this study, a detailed analysis connecting limiting conditions to the reliability of satellites for routine monitoring of CH_4_ emissions from global O&G targets had not been reported. Our analyses indicate that it is difficult to reliably monitor CH_4_ emissions from O&G infrastructure in countries outside the subtropics and dryland regions with TROPOMI and likely with other low Earth orbit (LEO) sun-synchronized passive satellite systems (see Supplementary Table S3) on a routine or on-demand basis. If large CH_4_ releases occur in Venezuela, for example, our results indicate a very low likelihood that TROPOMI and similar passive satellite systems will detect them. This could have several ramifications beyond the specific emissions events. First, our understanding of the spatial distribution of large emissions events based on satellite observations is incomplete^24^. CH_4_ emitted from undetected events may contribute substantially to the global CH_4_ budget. However, they will continue to escape satellite detection and reporting, causing uncertainties in emission estimates as undocumented sources. Second, as outlined earlier, some countries may become targeted by research and monitoring programs, including corollary media reports, owing to high observational coverage (e.g., Turkmenistan and Algeria), while others with low observational coverage are overlooked. These geographic disparities reflect the fact that more research and monitoring attention is likely to be directed at regions where the technology is more effective and where results can be published in the peer-review literature or media.

Low TOC over tropical and high latitude regions indicates these settings are less reliable for deriving satellite detections and quantifications. This includes broad swaths of Canada and Russia—two of the largest O&G producers globally. Many O&G producing countries in these regions experience prolonged periods (months) when there are no valid observations owing to persistent cloud cover and high SZA, and large emissions events, if present, can go undetected (see Supplementary Table S2). For emissions quantification, our results show that the majority of onshore O&G producing regions have low to moderate suitability scores. Hence, the accuracy of regional emissions estimates derived from satellite observations using the inversion method will vary between O&G producing regions. Occasional TROPOMI observations are still valuable for supporting posterior estimations and comparison with priors in regions with low TOC, but complementary measurements from other technologies, such as vehicle and aircraft systems, are needed to reconcile the emissions with high confidence.

Our analyses show that there is an abundance of regions globally with O&G targets and seasons when emissions, if present, will be hidden from passive satellite systems for extended periods of time. This presents a challenge for monitoring programs and resulting policies and actions to reduce emissions. With knowledge of limiting conditions, some O&G producers could strategically schedule activities so that large emissions resulting from intentional releases (e.g., blowdowns) are undetectable by satellites, like in winter at high latitudes or during monsoon seasons. This may be more challenging in the future with planned active satellite systems such as MERLIN, which will use a LiDAR sensor^27^, but other planned passive satellites are likely to encounter similar limitations.

On an annual basis, this study demonstrates that passive satellite monitoring of CH_4_ emissions from onshore O&G infrastructure should not be viewed as a form of global-scale continuous monitoring with the ability to acquire regular snapshots for detecting large emissions events or regional quantifications. At best, the analysis of TROPOMI data reveals that passive satellite monitoring is periodic and based on a global patchwork of high and low observational coverage. This is unlikely to change substantially with the addition of new passive satellite systems. In some regions, passive satellites like TROPOMI can provide high temporal coverage for detecting large emissions and quantifying rates from onshore O&G sources, but in many regions, they are only occasionally useful. Understanding the current spatial and temporal gaps of TROPOMI and the variables affecting its performance provides insight on similar challenges that forthcoming satellite systems may face. For example, the effect of cloud cover on TOC suggests that spatial resolution is a key consideration in the design of future satellites. Lack of observational coverage in instances where clouds are convective and distributed but spatially limited could be overcome by developing systems with finer spatial resolution. This would enable the identification and segmentation of different types of clouds within a given scene and improve the retrieval and attribution of CH_4_ emissions^39,40^.

Satellite remote sensing is often promoted as a leading component of a comprehensive CH_4_ emissions monitoring system, such as the UN’s Methane Alert and Response System (MARS) launched at COP27. However, the quality of the observations for detecting and quantifying CH_4_ emissions from O&G targets and other sources is not equal across the globe. Comprehensive monitoring programs must therefore incorporate a mix of other technologies and methods to fill gaps in satellite observational coverage, and to detect large events below the detection capabilities of satellites. In many regions, satellites using passing remote sensing will serve a very limited role in monitoring. With considerable reliance on satellites in international initiatives like MARS, it is imperative that the scope of monitoring approaches is expanded to include other leading technologies and methods.

## Methods

### Overview

To assess TROPOMI observational coverage (TOC), we calculated the mean proportion of the year with valid CH4 observations for TROPOMI. The valid observations of TROPOMI spanning three years were determined using the quality assurance value (*qa_value*) in TROPOMI level-2 data product. In this analysis, a TROPOMI day was defined as one day in which a given observation from TROPOMI had *qa_value* ≥ 0.5. Three-year average TOC was analyzed globally using calculated TROPOMI days of geographical grids from 2019 to 2021. Additionally, we investigated the global impacts of four TOC-reducing factors: cloud cover fraction, solar zenith angle, surface albedo (SWIR), and AOT (NIR). Moreover, we assessed the reliability of TROPOMI for monitoring emissions from global O&G sectors by extracting TOC of grids associated with O&G sectors. Finally, we conducted a suitability analysis to evaluate the potential suitability of using atmospheric inverse modeling to quantify the regional emission of global O&G producing regions.

### TROPOMI level-2 data product and data quality assurance flag

Sentinel-5P (TROPOMI)—a low earth orbit near polar sun-synchronous earth observation satellite—relies on passive remote sensing to map atmospheric methane columns on daily basis. It produces 13 or 14 netCDF data files each day for the level-2 data product (i.e., one data file per orbit). The pixel size of images is 7 km × 7 km for data processed before 06 August 2019 and 7 km × 5.5 km for data collected after 06 August 2019. In this study, we downloaded Sentinel-5P TROPOMI CH_4_ Level 2 data products from 01 January 2019 to 31 December 2021 from NASA's Goddard Earth Sciences Data and Information Services Center.

The TROPOMI level-2 data product uses the retrieval quality assurance flag (*qa_value*) to indicate whether pixels (XCH_4_—column averaged methane concentration) can be used to detect and quantify emissions. The *qa_value* is a continuous quality descriptor, varying between 0 and 1, depending on the instrumental viewing angle, content of atmospheric components, surface albedo, topography, etc.^41^. Briefly, a priori filtering screens feasible ground pixels for XCH_4_ retrieval. Pixels must meet several criteria including: above land, cloud free, normal instrument status, non-mountainous areas, etc. After filtering, pixel specific XCH_4_ retrievals are performed using atmospheric and TROPOMI’s Level 1B (radiance and irradiance measurements) input data. During the XCH_4_ retrieval, the pixels with unsuitable retrieval conditions are flagged indicating low data quality. The parameters that define the *qa_value* can be found in Table S1. As suggested by the data providers and previous studies, a minimum *qa_value* for a valid XCH_4_ measurement is 0.5^11,14,22,24,30,41^. This represents a XCH_4_ retrieved under cloud-free conditions and filtered for conditions with sufficient atmospheric reflectance (solar zenith angle ≤ 70°), viewing zenith angle (< 60°), smooth topography (1 standard deviation of surface elevation, ≤ 80 m within a 5-km radius), low absorption and scatter radiation from aerosols in the atmosphere (aerosol optical thickness in the near-infrared ≤ 0.3) and dark surface (surface albedo in shortwave infrared ≥ 0.02). It is important to note that the removal of TROPOMI observations with surface albedo lower than 0.02 will automatically remove any observations with water bodies over the surface, including observations over the ocean.

### TROPOMI day and TROPOMI observational coverage (TOC)

**Figure 6:**
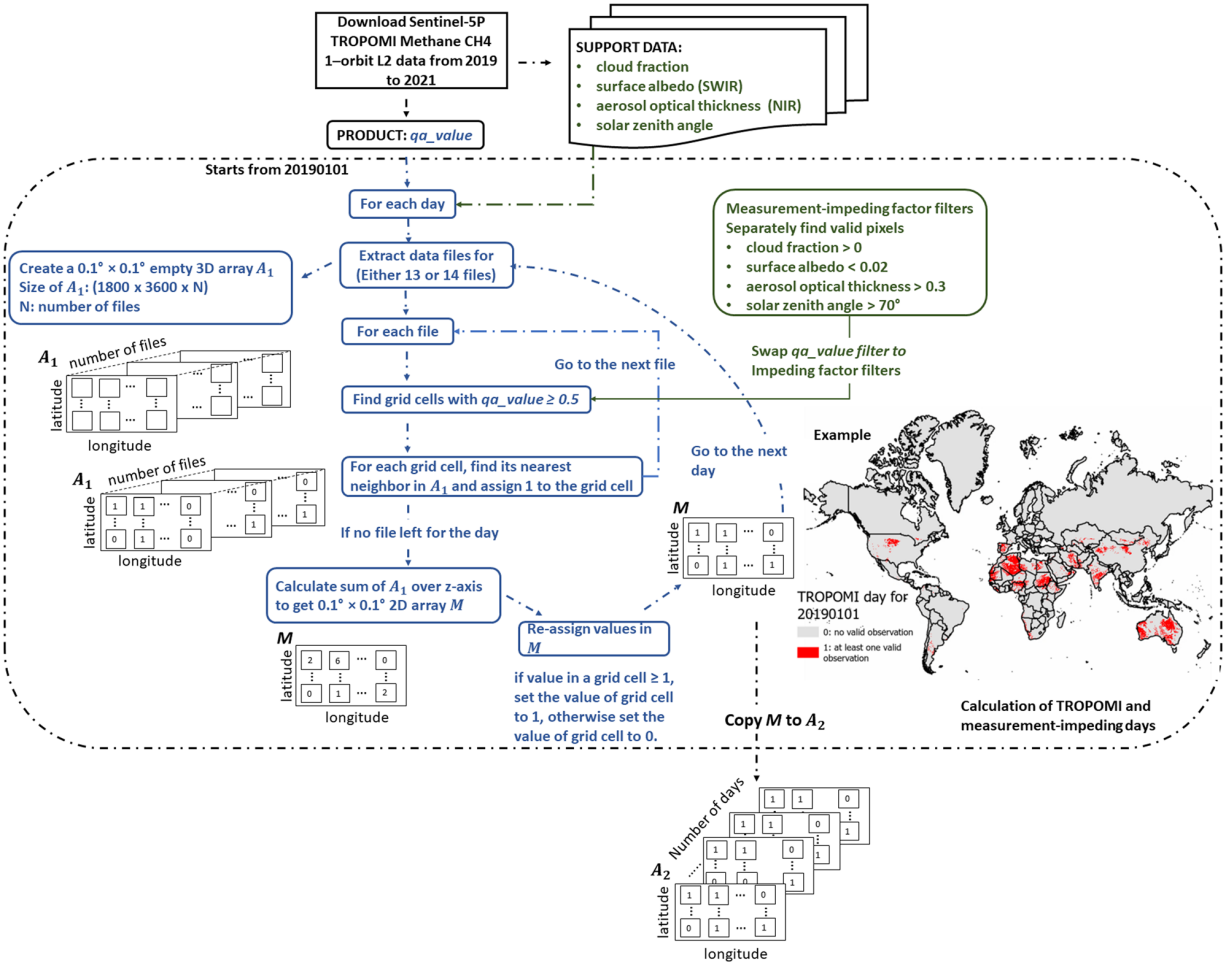
Workflow to calculate TROPOMI coverage.

In this study, we define a TROPOMI day as a pixel-covered area (hereinafter a "pixel") with at least one valid observation from TROPOMI (i.e., a XCH_4_ measurement with a *qa_value* ≥ 0.5) on a given day. The proportion of TROPOMI days per year for each pixel was then used to calculate the global TROPOMI observational coverage (TOC). To count TROPOMI days per year for each pixel, we constructed a workflow to sequentially determine if a given pixel and timestep combination satisfied the *qa_value* threshold (Fig. 6). We created two 3D global arrays, *A*_*1*_ and *A*_*2*_, to store the number of valid observations per day and TROPOMI days per year, respectively. Thus, the depth of *A*_*1*_ is either 13 or 14 (depending on the number of files per date-stamp), and the depth of *A*_*2*_ is 365 (366 for the year 2020). The column and row of both 3D arrays are longitude and latitude, respectively, and each pixel has a spatial resolution of 0.1° × 0.1°.

The daily workflow (Fig. 1) starts by finding all data files corresponding to the date ($d$). A quality assurance (QA) filter was used to find all valid pixels ($QA_{ij}$) for each file. Next, we found the nearest neighbor grid cells ($C_{ij}$) in *A*_*1*_ for all valid pixels by searching for the minimum Haversine distance between geographic coordinates of valid pixels and $C_{ij}$. Then, we assigned 1 to $C_{ij}$ to indicate one valid observation. This workflow was done recursively for all files. Following our definition of a TROPOMI day, we converted *A*_*1*_ to a 2D binary array (*M*). The grid cell of *M* with at least one valid observation indicates one TROPOMI day. At the end of the daily workflow, we assigned *M*–*A*_*2*_ based on the date-stamp. This daily workflow was repeated from 01 January 2019 to 31 December 2021. Finally, we summed *A*_*2*_ and divided it by the number of years of analysis ($n$ = 3) to find the average annual TROPOMI days per pixel. Then, we divided the average annual TROPOMI days by 365 and multiplied by 100 to calculate the average annual TOC ($TOC_{ij}$). The general formulas for calculating $TOC_{ij}$ are shown in Eqs. 1 and 2. In addition to average annual TOC, TROPOMI days were examined seasonally for all land-based grid cells. We also assessed average seasonal TOC for four seasons (01 September to 30 November, 01 December to 28/29 Feb, 01 March to 31 May, and 01 June to 31 August) to show the temporal variation in TROPOMI's data availability. This analysis was performed for 3 years from 2019 to 2021.

1 $$ TOC_{ij} = \left( {\frac{{\mathop \sum \nolimits_{1}^{n} \mathop \sum \nolimits_{1}^{365} C_{ij} }}{n}} \right) \div 365 \times 100 $$

where

2 $$ C_{ij} = \left\{ {\begin{array}{*{20}c} {0, QA_{ij} < 0.5} \\ {1, QA_{ij} \ge 0.5} \\ \end{array} } \right. $$

### Assessment of factors that reduce *TOC*

Cloud cover, SZA, viewing zenith angle, topography, AOT, and surface albedo in SWIR are six factors that can affect the quality of TROPOMI's observations and measurement of XCH_4_^30,31^. Except for topography, the other five factors are included in the TROPOMI level-2 data product and are measured by Sentinel-5P simultaneously when measuring XCH_4_ or from observations with co-located satellites (e.g., some cloud cover fraction from the L2 data product was measured from the Visible Infrared Imaging Radiometer Suite on the U.S. S-NPP). Since the current L2 TROPOMI data product already removed observations with viewing zenith angles > 60°^40^, we only quantified the effect of the remaining four factors on TOC according to the workflow described in Fig. 6. The goal was to determine how global variations in TOC are affected by each factor. First, we created filters to separately find pixels with cloud cover fraction > 0, SZA > 70°, surface albedo (SWIR) < 0.02, and AOT (NIR) > 0.3. When any one of these conditions is met, the observations are invalid because the *qa_value* < 0.5, so TOC cannot be derived according to Eq. 1 and 2. Second, we calculated the proportion of times per year for each factor that failed to meet its quality assurance requirement. This was performed separately for each factor, averaged for three years, and converted to a percentage. It is worth noting that the *qa_value* of a given grid cell and day combination can be affected by multiple factors. These factors also affect XCH_4_ retrieval for other passive remote sensing satellites. Therefore, investigating the impacts of different factors on TROPOMI also reveals potential limitations for other similar satellites.

### Impact of TOC on monitoring CH_4_ emissions from the O&G sector

We evaluated how TOC affects the ability to monitor CH_4_ emissions from the O&G sector. We used an inventory (0.1° × 0.1°) of O&G sector CH_4_ emissions (Fuel Exploitation Gas, Fuel Exploitation Oil, and Oil Refineries and Transformation Industry) from the Emissions Database for Global Atmospheric Research v6.0^42^ and excluded TOC of non-O&G grid cells in our analysis. We created a binary grid that denoted the presence or absence of onshore O&G activity to mask areas that had a high probability of containing O&G derived CH_4_ emissions. We also calculated the average consecutive TROPOMI days and average gap (i.e., number of days between two TROPOMI days) to further assess the continuity and observational gaps of using TROPOMI (or similar satellites) to monitor onshore O&G CH_4_ emissions in different countries. We focused our analysis on the top-10 O&G producing countries: USA, Russia, Saudi Arabia, Canada, Iraq, China, United Arab Emirates, Iran, Brazil, and Kuwait^35^.

Next, we evaluated the effect of TOC on regional CH_4_ emission quantification using TROPOMI observations. Atmospheric inverse modelling combined with Bayesian inference is the most widely used technique to estimate regional CH_4_ emissions with satellite observations^14,22,23,29^. The number of valid satellite observations, the accuracy of the bottom-up (BU) inventory data (also known as the prior), and uncertainty are three factors that can impact the confidence of a posterior estimation^6,14,20,23,43^. Accurate posterior estimates require an adequate number of valid observations to perform Bayesian inference. Therefore, error in the posterior estimate depends on errors in observations and the prior. Observational error has an inverse relationship with the number of observations; error is reduced as the number of observations increases. This indicates that TROPOMI (or similar satellites) are less suitable for monitoring CH_4_ emissions in regions with limited valid observations and large prior uncertainty. To identify these regions, we estimated the suitability score ($SS$) of using the inversion method to estimate CH_4_ emissions by integrating global TOC of O&G sector grid cells and the relative uncertainties ($RU$) of CH_4_ emissions in the BU inventory. The $SS$ was calculated using the following equation:

3 $$ SS = 1 - \left( {RU - RU \times TOC} \right) $$

where $RU$ is the relative standard deviation of the onshore O&G prior (between 0 and 100%) from the Global Fuel Exploitation Inventory v2^44^, which was created to improve the accuracy of inversion method and was evaluated by comparing to other BU inventories in the previous studies^44^. As such, the high $RU$ identifies regions with large uncertainty with respect to inventory of CH_4_ emission. The value of $SS$ also lies between 0 and 1. Regions with $SS$ close to 1 are regions suitable for inversion method, which occurs when $RU$ is close to 0 (low relative uncertainty), TOC is close to 1 (high observational coverage from TROPOMI), or both.

### Supplementary Information

Supplementary Information.

## Data Availability

The datasets analyzed during the study, if not in the Supplementary Information, are available from the corresponding author on reasonable request.

## Code availability

The TROPOMI observational coverage processing and analysis were programmed in Python with standard packages. The scripts can be found in https://github.com/MozhouGao/Global-TROPOMI-Observatioanl-Coverage. Data required to perform TROPOMI observational coverage calculation can be downloaded from either Copernicus Open Access Hub or NASA Goddard Earth Science Data and Information Services Center. The results can be reproduced by employing the equations, explanation, and parameters provided in the main text. Additional code and data will be made available upon request.
