# Supplementary material for: Global observational coverage of onshore oil and gas methane sources with TROPOMI
Source: Sci Rep. 2023 Oct 5;13:16759. doi: 10.1038/s41598-023-41914-8 (PMC10555993; doi:10.1038/s41598-023-41914-8)
Supplement: Supplementary file 1 — Supplementary Information. [file 41598_2023_41914_MOESM1_ESM.docx]

*Supplementary information of*

**Global observational coverage of onshore oil and gas methane sources with TROPOMI**

Mozhou Gao^1*^, Zhenyu Xing^1^, Coleman Vollrath^1^, Chris H. Hugenholtz^1^, Thomas E. Barchyn^1^

^1^Centre for Smart Emissions Sensing Technologies, Department of Geography, University of Calgary, 2500 University Drive NW, Calgary, AB, T2N 1N4, Canada

*Correspondence to: Mozhou Gao ([mozhou.gao@ucalgary.ca](mailto:mozhou.gao@ucalgary.ca))


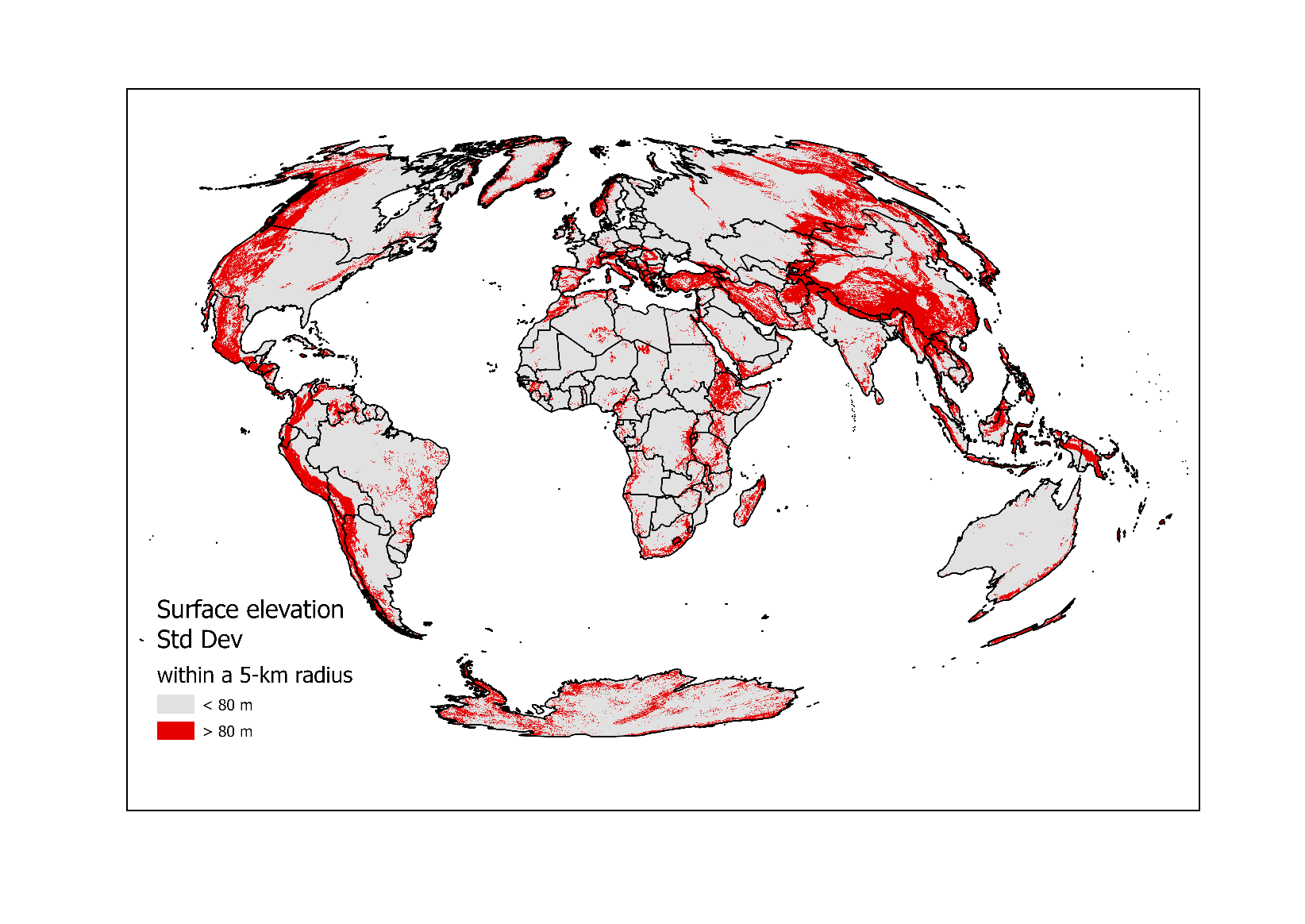


**Figure S1.** Map shows regions with standard deviations of surface elevation >80 m within a 5-km radius. The standard deviations were calculated by using Global Land One-kilometer Base Elevation dataset [1]. The map was created using ArcGIS Pro 3.1.2 (https://www.esri.com/en-us/arcgis/products/arcgis-pro/overview).


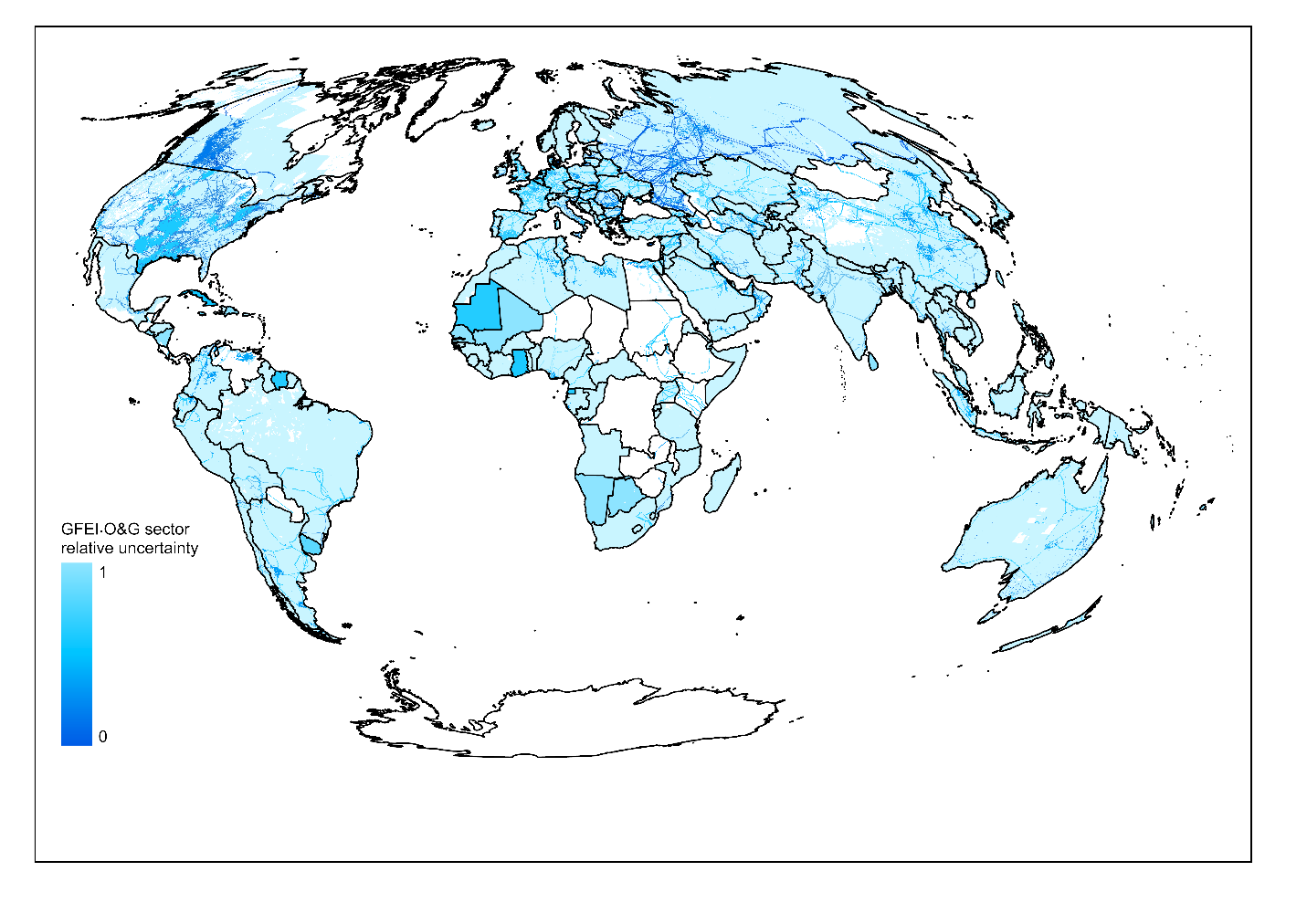

**Figure S2.** Relative uncertainty of the Global Fuel Exploitation Inventory (GFEI) for the O&G sector [2]. The map was created using ArcGIS Pro 3.1.2 (https://www.esri.com/en-us/arcgis/products/arcgis-pro/overview).

**Table S1.** Definition of the quality assurance flag (qa_value) for TROPOMI’s XCH_4_ retrieval [3].

| qa_value | Condition |
| --- | --- |
| 1.0 | Convergence, clear-sky |
| 0.8 | Failed deconvolution irradiance spectrum |
| 0.4 | - Not confidentially clear-sky (VIIRS, non-scattering retrieval as back-up) - SZA > 70° - Surface albedo (SWIR) < 0.02 - AOT (NIR) > 0.3 - CH_4_ noise related error > 10 χ2 > 100 - Terrain roughness > 80 |
| 0.0 | No convergence or Pre-filter:   - Cloud fraction > 0.02 - Terrain roughness > 100 m - SZA > 75°, VZA > 60° - Fraction of “good” spectral pixels (SIWR, NIR) < 70% - SNR SWIR > 50 |

**Table S2.** 3-year average TROPOMI observational coverages, 3-year average gap TROPOMI days, and 3-year average consecutive TROPOMI days for O&G sector grid cells of 160 countries.

| **Descriptions** |
| --- |
| CID: Unique ID for each country |
| TOC:3-year average annual TROPOMI observational coverage (%) |
| Gaps:3-year average gap TROPOMI days |
| Consecutives:3-year average consecutive TROPOMI days |

GMP: whether a partner of Global Methane Pledge

| CID | Country | TOC (%) | Gaps | Consecutives | GMP |
| --- | --- | --- | --- | --- | --- |
| 0 | Afghanistan | 10 | 24 | 2 | N |
| 1 | Albania | 0 | 365 | 0 | Y |
| 2 | Algeria | 23 | 7 | 3 | N |
| 3 | Angola | 7 | 25 | 2 | N |
| 4 | Argentina | 13 | 12 | 2 | Y |
| 5 | Armenia | 0 | 365 | 0 | Y |
| 6 | Australia | 6 | 25 | 2 | N |
| 7 | Austria | 5 | 28 | 1 | N |
| 8 | Azerbaijan | 1 | 207 | 0 | N |
| 9 | Bahrain | 0 | 365 | 0 | Y |
| 10 | Bangladesh | 8 | 21 | 2 | N |
| 11 | Belarus | 4 | 39 | 2 | N |
| 12 | Belgium | 6 | 24 | 2 | Y |
| 13 | Benin | 2 | 64 | 2 | Y |
| 14 | Bolivia | 4 | 101 | 1 | N |
| 15 | Bosnia | 0 | 238 | 0 | Y |
| 16 | Brazil | 2 | 90 | 1 | Y |
| 17 | Brunei Darussalam | 0 | 365 | 0 | N |
| 18 | Bulgaria | 7 | 22 | 2 | Y |
| 19 | Burkina Faso | 12 | 12 | 3 | Y |
| 20 | Burundi | 0 | 192 | 0 | N |
| 21 | Côte d'Ivoire | 1 | 111 | 1 | N |
| 22 | Cambodia | 0 | 255 | 0 | N |
| 23 | Cameroon | 1 | 186 | 1 | Y |
| 24 | Canada | 4 | 31 | 2 | Y |
| 25 | Canarias | 0 | 365 | 0 | N |
| 26 | CAR | 1 | 199 | 1 | Y |
| 27 | Chad | 12 | 13 | 3 | N |
| 28 | Chile | 2 | 78 | 1 | Y |
| 29 | China | 5 | 42 | 1 | N |
| 30 | Colombia | 0 | 284 | 0 | Y |
| 31 | Comoros | 0 | 365 | 0 | N |
| 32 | Congo | 0 | 304 | 0 | N |
| 33 | Congo DRC | 1 | 105 | 1 | N |
| 34 | Costa Rica | 0 | 365 | 0 | Y |
| 35 | Croatia | 10 | 13 | 2 | Y |
| 36 | Cuba | 1 | 93 | 1 | Y |
| 37 | Cyprus | 0 | 365 | 0 | Y |
| 38 | Czech Republic | 4 | 33 | 2 | N |
| 39 | Denmark | 2 | 56 | 1 | Y |
| 40 | Djibouti | 8 | 30 | 2 | Y |
| 41 | Dominican Republic | 0 | 294 | 0 | Y |
| 42 | Ecuador | 0 | 284 | 0 | Y |
| 43 | Egypt | 14 | 15 | 2 | N |
| 44 | El Salvador | 0 | 365 | 0 | Y |
| 45 | Equatorial Guinea | 0 | 365 | 0 | N |
| 46 | Eritrea | 7 | 18 | 2 | N |
| 47 | Estonia | 2 | 70 | 1 | Y |
| 48 | Eswatini | 18 | 8 | 3 | N |
| 49 | Ethiopia | 1 | 162 | 0 | Y |
| 50 | Finland | 1 | 81 | 1 | Y |
| 51 | France | 6 | 27 | 2 | Y |
| 52 | Gabon | 0 | 365 | 0 | Y |
| 53 | Gambia | 3 | 189 | 0 | Y |
| 54 | Georgia | 0 | 365 | 0 | Y |
| 55 | Germany | 5 | 26 | 2 | Y |
| 56 | Ghana | 1 | 111 | 1 | Y |
| 57 | Greece | 0 | 365 | 0 | Y |
| 58 | Guatemala | 0 | 365 | 0 | Y |
| 59 | Guinea | 5 | 27 | 2 | N |
| 60 | Guinea Bissau | 16 | 9 | 3 | N |
| 61 | Haiti | 0 | 365 | 0 | N |
| 62 | Honduras | 0 | 365 | 0 | Y |
| 63 | Hungary | 8 | 16 | 2 | N |
| 64 | India | 12 | 15 | 3 | N |
| 65 | Indonesia | 0 | 304 | 0 | Y |
| 66 | Iran | 6 | 35 | 2 | N |
| 67 | Iraq | 22 | 8 | 3 | Y |
| 68 | Ireland | 1 | 75 | 1 | Y |
| 69 | Israel | 9 | 30 | 2 | Y |
| 70 | Italy | 5 | 34 | 1 | Y |
| 71 | Jamaica | 0 | 365 | 0 | Y |
| 72 | Japan | 1 | 90 | 0 | Y |
| 73 | Jordan | 14 | 18 | 2 | Y |
| 74 | Kazakhstan | 13 | 11 | 2 | N |
| 75 | Kenya | 1 | 131 | 0 | N |
| 76 | Kuwait | 37 | 4 | 3 | Y |
| 77 | Kyrgyzstan | 0 | 365 | 0 | Y |
| 78 | Laos | 2 | 50 | 2 | N |
| 79 | Latvia | 4 | 30 | 2 | N |
| 80 | Lebanon | 0 | 365 | 0 | N |
| 81 | Lesotho | 12 | 11 | 2 | N |
| 82 | Liberia | 2 | 103 | 2 | Y |
| 83 | Libya | 22 | 7 | 3 | Y |
| 84 | Liechtenstein | 0 | 243 | 0 | Y |
| 85 | Lithuania | 4 | 32 | 2 | N |
| 86 | Luxembourg | 6 | 21 | 2 | Y |
| 87 | Madagascar | 5 | 26 | 2 | N |
| 88 | Malawi | 5 | 22 | 2 | Y |
| 89 | Malaysia | 0 | 365 | 0 | Y |
| 90 | Mali | 9 | 19 | 2 | Y |
| 91 | Mauritania | 3 | 191 | 0 | N |
| 92 | Mexico | 5 | 27 | 2 | Y |
| 93 | Moldova | 9 | 15 | 2 | N |
| 94 | Mongolia | 5 | 26 | 2 | N |
| 95 | Morocco | 15 | 14 | 2 | Y |
| 96 | Mozambique | 4 | 36 | 2 | N |
| 97 | Myanmar | 5 | 29 | 2 | N |
| 98 | Namibia | 18 | 10 | 3 | N |
| 99 | Netherlands | 5 | 30 | 2 | Y |
| 100 | NewZealand | 0 | 271 | 0 | Y |
| 101 | Nicaragua | 0 | 365 | 0 | N |
| 102 | Niger | 11 | 18 | 2 | N |
| 103 | Nigeria | 2 | 77 | 1 | Y |
| 104 | North Korea | 2 | 71 | 1 | N |
| 105 | North Macedonia | 5 | 30 | 2 | Y |
| 106 | Norway | 0 | 365 | 0 | Y |
| 107 | Oman | 5 | 35 | 2 | N |
| 108 | Pakistan | 12 | 17 | 2 | Y |
| 109 | Palau | 0 | 365 | 0 | Y |
| 110 | Palestinian Territory | 8 | 19 | 2 | N |
| 111 | Panama | 0 | 365 | 0 | Y |
| 112 | Paraguay | 12 | 13 | 2 | N |
| 113 | PEG | 0 | 365 | 0 | Y |
| 114 | Peru | 0 | 365 | 0 | Y |
| 115 | Philippines | 0 | 365 | 0 | Y |
| 116 | Poland | 5 | 27 | 2 | N |
| 117 | Portugal | 6 | 140 | 1 | Y |
| 118 | Qatar | 3 | 50 | 1 | Y |
| 119 | Romania | 5 | 28 | 2 | N |
| 120 | Russian Federation | 4 | 37 | 2 | N |
| 121 | Rwanda | 0 | 223 | 0 | Y |
| 122 | San Marino | 0 | 365 | 0 | N |
| 123 | Saudi Arabia | 21 | 8 | 3 | Y |
| 124 | Senegal | 12 | 13 | 2 | Y |
| 125 | Serbia | 7 | 24 | 2 | Y |
| 126 | Sierra Leone | 3 | 39 | 2 | N |
| 127 | Singapore | 0 | 365 | 0 | Y |
| 128 | Slovakia | 3 | 46 | 1 | N |
| 129 | Slovenia | 3 | 36 | 1 | Y |
| 130 | Somalia | 6 | 34 | 2 | N |
| 131 | South Africa | 12 | 13 | 2 | N |
| 132 | South Korea | 3 | 31 | 1 | Y |
| 133 | South Sudan | 6 | 19 | 2 | N |
| 134 | Spain | 3 | 77 | 1 | Y |
| 135 | SriLanka | 0 | 365 | 0 | N |
| 136 | Sudan | 18 | 9 | 3 | N |
| 137 | Suriname | 0 | 243 | 0 | Y |
| 138 | Sweden | 1 | 284 | 0 | Y |
| 139 | Switzerland | 1 | 162 | 0 | Y |
| 140 | Syria | 28 | 6 | 3 | N |
| 141 | Tajikistan | 0 | 365 | 0 | N |
| 142 | Tanzania | 1 | 82 | 1 | N |
| 143 | Thailand | 4 | 54 | 1 | N |
| 144 | Togo | 3 | 46 | 1 | Y |
| 145 | Tunisia | 12 | 11 | 2 | Y |
| 146 | Turkey | 2 | 105 | 1 | N |
| 147 | Turkmenistan | 20 | 9 | 3 | N |
| 148 | UAE | 9 | 21 | 2 | Y |
| 149 | Uganda | 0 | 148 | 0 | N |
| 150 | Ukraine | 6 | 27 | 2 | Y |
| 151 | United Kingdom | 2 | 53 | 1 | Y |
| 152 | United States | 10 | 15 | 2 | Y |
| 153 | Uruguay | 14 | 10 | 2 | Y |
| 154 | Uzbekistan | 16 | 11 | 3 | Y |
| 155 | Venezuela | 0 | 304 | 0 | N |
| 156 | Vietnam | 1 | 182 | 1 | Y |
| 157 | Yemen | 6 | 19 | 2 | N |
| 158 | Zambia | 9 | 18 | 2 | Y |
| 159 | Zimbabwe | 15 | 12 | 3 | N |

**Table S3.** Current and planned satellite instruments for observing atmospheric CH_4_

| Satellite instruments | Launch time (or planned launch time) | Passive or active remote sensing | Orbit Type | Orbit altitude (km) |
| --- | --- | --- | --- | --- |
| GOSAT | 2009 | passive | Sun-synchronous | 666 |
| Landsat-8 | 2013 | passive | Sun-synchronous | 705 |
| WorldView-3 | 2014 | passive | Sun-synchronous | 617 |
| Sentinel-2 | 2015 | passive | Sun-synchronous | 786 |
| GHGSats | 2016 | passive | Sun-synchronous | 512-550 |
| TROPOMI | 2017 | passive | Sun-synchronous | 824 |
| Gaofen3 | 2018 | passive | Sun-synchronous | 755 |
| PRISMA | 2019 | passive | Sun-synchronous | 614 |
| Ziyuan1 | 2019 | passive | Sun-synchronous | 785 |
| EnMAP | 2022 | passive | Sun-synchronous | 653 |
| EMIT | 2022 | passive | Sun-synchronous | 400 |
| GOSAT-GW | 2023 | passive | Sun-synchronous | 667 |
| MethaneSAT | 2023 | passive | Sun-synchronous | NA |
| MicroCarb | 2023 | passive | Sun-synchronous | 650 |
| Carbon Mapper | 2023 | passive | Sun-synchronous | NA |
| Sentinel-2C | 2024 | passive | Sun-synchronous | NA |
| GeoCarb | 2025 | passive | Geostationary | 35786 |
| CO2M | 2025 | passive | Sun-synchronous | NA |
| MERLIN | 2027 | active | Sun-synchronous | 500 |

**References**

1. Hastings, D. & Dunbar, P. K. Global Land One-kilometer Base Elevation (GLOBE) v.1. National Geophysical Data Center, NOAA (1999). https://www.ncei.noaa.gov/access/metadata/landing-page/bin/iso?id=gov.noaa.ngdc.mgg.dem:280 (Accessed 07 March 2023).

2. Scarpelli, T. R. *et al.* Global Inventory of Methane Emissions from Fuel Exploitation. Goddard Earth Sciences Data and Information Services Center (GES DISC) (2021). https://disc.gsfc.nasa.gov/datasets/GFEI_CH4_1/summary (Accessed 07 March 2023).

3. Landgraf, J. *et al.* S5P Mission Performance Centre Methane [L2__CH4___] Readme (2022).
